# Hallucination Proneness is Linked to Over-Reliance on Internal Priors for Noisy Speech

**DOI:** 10.1093/schbul/sbag048

**Published:** 2026-05-09

**Authors:** Izabele Batkovskyte, Naresh Subramaniam, Johanna Finnemann, Matthew H Davis, Paul C Fletcher

**Affiliations:** Department of Psychiatry, University of Cambridge, Cambridge, CB2 0SZ, United Kingdom; Department of Psychiatry, University of Cambridge, Cambridge, CB2 0SZ, United Kingdom; Department of Psychiatry, University of Cambridge, Cambridge, CB2 0SZ, United Kingdom; Max Planck Institute for Human Cognitive and Brain Sciences, Leipzig, 04303, Germany; Medical Research Council Cognition and Brain Sciences Unit, University of Cambridge, Cambridge, CB2 7EF, United Kingdom; Department of Psychiatry, University of Cambridge, Cambridge, CB2 0SZ, United Kingdom

**Keywords:** psychosis, schizotypy, predictive processing, perception, source monitoring

## Abstract

**Background:**

According to predictive processing models, hallucinations can arise from over-weighting of prior expectations relative to sensory input. Individuals who hallucinate may misattribute internally-generated experiences to external sources. Combining these mechanisms, we hypothesized that hallucination-proneness in a non-clinical sample is associated with a greater reliance on internally-generated priors compared to externally-provided priors during degraded speech perception.

**Study Design:**

Two online experiments were conducted with healthy adult participants. Experiment 1 (*n* = 92) targeted lower-level perceptual processes by assessing sensitivity and bias in distinguishing regular vocoded speech from unintelligible, spectrally-rotated vocoded stimuli at varying levels of sensory clarity. Experiment 2 (*n* = 100) compared the influence of internally-generated versus externally-provided priors on perception of vocoded speech content. Internal- and external-priors were matched in reliability to test if hallucination-proneness was linked to differential influences of internally-generated priors on speech perception.

**Study Results:**

Experiment 1 confirmed that participants who are more hallucination-prone showed a reduced ability to distinguish potentially intelligible speech from unintelligible speech, and a higher bias toward indicating that speech was present in unintelligible stimuli. Experiment 2 confirmed differential effects of internal priors, showing that hallucination-proneness scores were linked to a greater influence on internally-generated priors on perceptual report when these were incorrect, and a lower reliance on externally-provided priors when these were correct.

**Conclusions:**

These findings suggest that hallucination-proneness is linked to an increased influence of internally-generated compared to externally-provided predictions on perception, suggesting an additional element of predictive processing theories of hallucinations.

## Introduction

Within predictive processing models,^1-4^ hallucinations—that is, percepts in the absence of stimuli—are theorized to arise from an overweighting of prior expectations relative to sensory evidence.^1^^,^^5-7^ Empirical support for this idea comes from studies in both clinical and non-clinical populations where hallucinations and aberrant perceptual experiences are associated with an increased reliance on prior knowledge during perceptual inference.^2^^,^^8-11^

While use of prior expectations in perception is universal to all sensory modalities, auditory studies are particularly important since auditory-verbal hallucinations are so prominent in psychosis. Conditioned-hallucination paradigms provide consistent evidence that hallucination-proneness is linked to heavier weighting of implicitly learned, task-provided priors across multiple samples.^2^^,^^7^^,^^12^^,^^13^ Some studies suggest that hallucination-proneness may reflect a general prior expectation to hear speech in noisy signals, rather than an enhanced sensitivity to experimentally induced priors,^14^^,^^15^ while studies using tasks that explicitly manipulate prior precision through semantic or lexical expectations show that higher schizotypy is associated with a stronger influence of high-level priors, even when those conflict with sensory input.^16^ These apparently contrasting findings may partly reflect differences in prior source—for example, externally provided, experimenter-defined priors^15^ versus internally generated semantic predictions arising from sentence context.^16^

The influence of prior expectations in generating hallucinations may therefore depend on both the level of perceptual processing and the source of the prior. Neural accounts of speech perception—whether predictive or not—are inherently hierarchical, with lower levels processing basic auditory features to determine overall properties of a sound (eg, whether a sound is speech or not) while higher levels support more abstract inferences, such as identifying the words spoken and their intended meaning.^17-19^ Predictive processing theories further suggest a hierarchy of top-down predictions such that auditory processing is influenced by prior expectations for the perceptual form and content of speech.^20-22^ In psychosis, precision of priors may be abnormally distributed across this hierarchy—reduced at lower levels, and increased at higher ones, leading to disproportionate reliance on abstract, internally-generated priors for words and meanings over sensory input from the environment.^3^^,^^4^^,^^23^

Perceptual inference also depends on accurate source monitoring—the ability to correctly identify whether a given stimulus originates from internal sources, such as memory or imagination, or external ones, such as the environment.^24^ Source monitoring impairments in psychosis are well documented—individuals with hallucinations are more likely to misattribute internally generated experiences to external sources, and show reduced activation in prefrontal brain regions linked to successful source monitoring.^25-31^ This may reflect inaccurate estimations of the precision of different sources of information—individuals prone to hallucinations may consider internally-generated priors more precise than externally provided ones, particularly when sensory signals are ambiguous or degraded.

To investigate these questions, we designed two tasks that involved perception of degraded speech targeting different levels of the perceptual hierarchy. The first task (Experiment 1) targeted lower-level processes involved in identifying speech, as in earlier studies.^15^^,^^32^ We assessed participants’ sensitivity and bias in distinguishing somewhat intelligible degraded speech from always unintelligible forms of degraded speech as sensory clarity increased. The second task (Experiment 2) targeted higher-level inferences about which words are heard in degraded speech by assessing perception under conditions in which participants have strong prior expectations for which word is likely to be heard. In this second task, we compared the influence of internally-generated (from participant’s own guesses of which word they were hearing) versus externally-provided priors (generated by the experimenter) on perception of speech content. To examine whether hallucination-proneness in a nonclinical sample was associated with a differential weighting of these 2 types of priors, we ensured that both priors were equally informative: matching the subsequent word 50% of the time. Comparing perception in these conditions allowed us to test the hypothesis that hallucination-proneness is specifically linked to overweighting of internally-generated prior predictions.

## Experiment 1

### Methods

#### Participants

Ninety-two (46 female) participants were recruited from Prolific Academic. All self-reported to be native monolingual English speakers, aged 19-77 years (M(SD), 47.80(12.63)) and to have no hearing impairment, language-related disorders, or psychiatric diagnosis. Participants received £7 for their time (~£6 per hour). Informed consent was obtained via Qualtrics, under a process approved by the Cambridge Psychology Research Ethics Committee.

#### Stimuli

We selected 100 highly frequent and confusable monosyllabic English words from the CELEX database.^33^ Words selected were 3 or more phonemes in length, had a frequency of occurrence greater than 100/million, and chosen to have large numbers of phonological neighbors (ie, many other words in the CELEX database can be made with a single phoneme addition, substitution, or deletion). We selected the 100 words with the highest number of phonological neighbors, which ranged from 27 to 43. These words were synthesized using Google Text-To-Speech, using a female British English voice (https://cloud.google.com/text-to-speech/, accessed July 17, 2025). The full list of spoken words is provided in Supporting Materials, S3.1.

**Figure 1:**
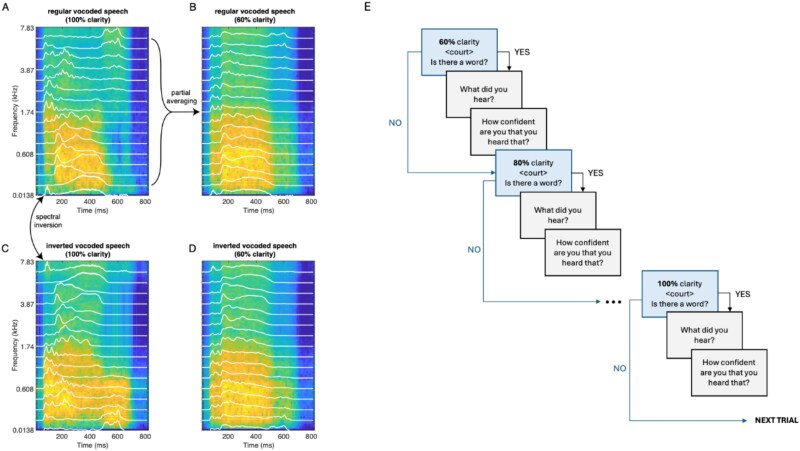
Left: example stimulus materials for the word “cause.” (A) Spectrogram of regular vocoded speech in the 100% clarity condition. This is degraded speech that remains highly intelligible for most listeners. White lines show 16 independent amplitude envelopes derived from the original speech recording, which are then used to modulate log-spaced bands of noise between 50 and 8000 Hz to create the vocoded stimulus. (B) Spectrogram of regular vocoded speech at 60% clarity. This is more severely degraded and hence unintelligible for most listeners. This condition is created by partially averaging each of the 16 individual amplitude envelopes with a single broadband envelope (ie, the mean of the 16 envelopes at 100% clarity). (C) Spectrogram of inverted vocoded speech at 100% clarity. This is a stimulus with the same spectral properties as regular vocoded speech, but that sounds entirely unlike speech. This condition is created by swapping high- and low-frequency amplitude envelopes in regular vocoded speech. (D) Spectrogram of inverted vocoded speech at 60% speech. Due to spectral averaging, this stimulus is difficult to distinguish from regular speech at 60% clarity. Example vocoded stimuli can be found at: https://doi.org/10.17605/OSF.IO/3KPMA. Right: Task procedure (E) each trial starts with a presentation of an inverted or a regular word at 60% clarity. If participants indicate that they can hear a word, they are asked to type what they heard and report their confidence in their percept. They then heard a second presentation of the same word at 80% clarity, followed by typed responses and confidence ratings as appropriate. This procedure is repeated at all 5 (60%, 80%, 90%, 95%, and 100%) clarity levels when they are prompted to proceed to the next trial.

The synthesized words were degraded using a morphed noise-vocoder based on previously described methods.^34^^,^^35^ Two types of vocoded stimuli were produced—regular and inverted. Regular stimuli were produced by extracting amplitude profiles (envelopes) from the speech signal within 16 logarithmically-spaced frequency bands, and superimposing these envelopes onto white noise filtered into the same 16 frequency bands. Spectral detail was varied by increasing the degree of similarity of these envelopes based on a parameter that determined the degree to which the 16 individual envelopes were mixed with a broadband (1-channel) envelope. With 100% mixing, all 16 envelopes remained unmodified, producing 16-channel vocoded speech, which is highly intelligible (Figure 1A). With 0% mixing, all 16 envelopes were identical to the broadband envelope, producing 1-channel vocoded speech, which is entirely unintelligible. This procedure is equivalent to morphing 16-channel and 1-channel vocoded speech in different proportions. We produced 5 regular stimuli for each word by varying the mixing percentage or clarity of 16-channel speech (60%, 80%, 90%, 95%, and 100%—referred to hereafter as *clarity levels*).

Inverted stimuli were produced by swapping the amplitude envelopes between the high- and low-frequency ranges before superimposing them onto band-pass filtered noise. This creates stimuli that retain spectral detail in a form that resembles speech but doesn’t support perception of words or identification of stimuli as being speech.^36^ Spectral detail was varied in the same way as for regular stimuli by morphing 16- and 1-channel envelopes in varying proportions. This resulted in 5 inverted stimuli for each word, corresponding to clarity levels of 60%, 80%, 90%, 95%, and 100%. At 100%, inverted stimuli are unintelligible and do not resemble human speech sounds. The complete stimulus set consisted of 1000 unique stimuli—100 synthesized monosyllabic words vocoded at 5 clarity levels for regular and inverted speech. Example spectrograms of regular and inverted vocoded stimuli are shown in Figure 1A,B and C,D respectively.

Each word was randomly assigned to the inverted or regular condition for each participant, so that participants heard 50 regular and 50 inverted words. Each word was presented once at 5 gradually increasing clarity levels, so that unique tokens were heard in all trials.

Before starting the task, participants heard a single example word in the inverted condition at 100% clarity, and regular conditions at 80% and 100% clarity, alongside the task instructions. The experimental task was programmed using jsPsych^37^ and hosted on a JATOS server^38^ at the MRC CBU. Participants accessed the task via links from Prolific Academic (https://www.prolific.com) and were encouraged to complete it in a quiet environment using headphones.

#### Task Procedure

The task consisted of 100 trials. Each trial consisted of 5 presentations of the same word, at 5 increasing levels of clarity. Participants were told they would hear various distorted sounds that may or may not contain a real monosyllabic English word. After each stimulus presentation, participants were asked “is there a word”. If they answered “no,” they heard the next stimulus in that trial, now at the next level of clarity, and were asked to indicate again if they thought a word was present.

If they answered “yes,” they were asked to type the word they heard. They were then asked to indicate how confident they were about their guess, using a slider (0-100 rating). They then heard the next stimulus.

This procedure was repeated for all 5 versions of each word presented in order of clarity (ie, regular and inverted words were presented first in their least clear form). Participants were then instructed to press any key on the keyboard to move to the next trial. The procedure of a single trial is shown in Figure 1E.

#### Questionnaires

Two questionnaires were administered on Qualtrics before the experimental task began: (1) the Cardiff Anomalous Perceptions Scale^39^ (CAPS) and (2) the Peters Delusion Inventory^40^ to measure hallucination-proneness and delusion-proneness, respectively. An attention-check item (“select ‘no’ for this item”) was included to detect careless responding; which all participants responded to correctly.

### Analysis

#### Outcome Measures

Main outcome measures were derived from signal detection theory^41^: 1) sensitivity (d’), measuring the ability to discriminate the 2 types of stimuli by responding “yes” to regular stimuli (hits), while avoiding false alarms (responding “yes” to inverted stimuli); and (2) criterion (C), a measure of participants’ bias toward reporting that they didn’t hear a word (positive values) or did hear a word (negative values).

In addition to scoring the ability to discriminate between regular and inverted stimuli, the accuracy of participants’ typed responses was quantified using a Levenshtein Distance ratio similar to that used in previous studies.^42^ Participants’ typed responses to each stimulus, and stimulus words were transcribed into phonemes using the DISC transcriptions from the CELEX database.^33^ A Levenshtein Ratio (LR) was then used calculated to measure percept accuracy^43^; a measure of the similarity between the 2 strings and hence the proportion of phonemes identified correctly. This measure gives partial credit to segments identified correctly, but in incorrect positions in the string.

### Results

#### CAPS Scores

Cardiff Anomalous Perceptions Scale scores had a mean of 37.2 (SD = 43.0) and a median of 21, with a positively skewed distribution. To optimize statistical power, we used a median split to produce 2 groups with high and low CAPS scores (See Supplement S1.3 for distribution plots and correlations with PDI scores).

### Overall Task Performance

We initially assessed how accuracy varied across clarity levels. These analyses showed expected effects of condition (regular/inverted), and clarity on task performance in that participants were more likely to judge that speech was present in regular trials, and this difference between regular and inverted speech increased in higher-clarity trials (see Supplement S1.1, Figure 1A). Moreover, participants’ accuracy at identifying spoken words increased with increasing clarity (see Supplement S1.1, Figure 1B which shows effects of clarity on the proportion of fully correct reports and on word report accuracy (Supplement S1.1, Figure 1C)).

**Figure 2:**
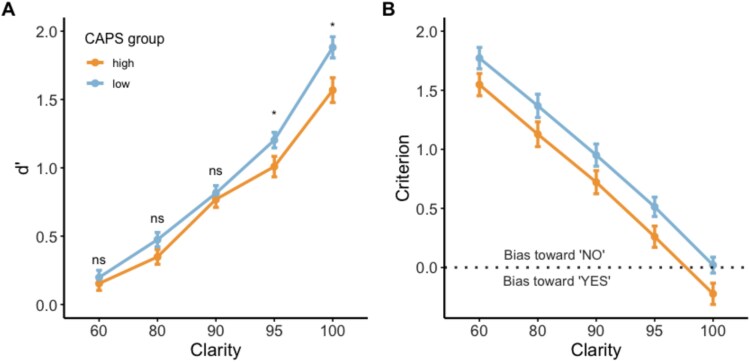
(A) Sensitivity (d’) to report that words are present for high- and low-CAPS groups *(mean* ± SEM) at each clarity level. Asterisks indicate significant pairwise differences between the 2 groups (^*^: *P* < .05, Bonferroni-adjusted). (B) Criterion values measuring bias toward responding “no” to the question “is there a word”. The dotted horizontal line represents optimal (unbiased) responding. Values above the line indicate that participants were biased toward responding “no,” and those below the dotted line indicate a bias toward responding “yes” statistical analysis showed a main effect of CAPS group, but no interaction.

We used signal detection measures to quantify participant’s ability to discriminate (sensitivity, d’) and response bias (criterion, C) when distinguishing regular and inverted speech. A one-way repeated-measures analysis of variance (ANOVA) showed significant differences in d’ between the different clarity levels, F(2.84258.22) = 343.67, *P* < .0001, η^2^*_G_* = 0.61, 95% CI, 0.56,1.00 (Greenhouse–Geisser corrected; Supplement S1.1, Figure 2A). Criterion values were positive, indicating that participants were biased toward reporting that they didn’t hear spoken words. The mean criterion across all participants and clarity levels was significantly greater than zero (M = 0.723, t(91) = 13.477, *P* < .0001, 95% CI, 0.617;0.830), indicating that despite regular speech being present in 50% of trials, participants showed an overall bias toward reporting that they did not hear speech. Criterion values were also significantly different between different clarity levels, F(2.15, 196.1) = 623.09, *P* < .0001, η^2^*_G_* = 0.496, 95% CI, 0.44;1.00 (Supplement S1.1, Figure 2B).

#### Effects of Hallucination-Proneness on Perceptual Performance

We investigated differences in perceptual performance between low-CAPS and high-CAPS individuals based on a median split over all participants. We report results on the sensitivity to distinguish regular and inverted speech (d’, Figure 2A), and bias toward/away from reporting that words are present (Criterion, Figure 2B) in high- and low-CAPS individuals. Differences in word report accuracy between high- and low-CAPS are reported in Supplementary Materials S1.2.

#### Sensitivity to Detect Speech (d’)

A two-way mixed ANOVA showed statistically significant main effects of CAPS group (high/low) (F(1, 90) = 4.403, *P* = .0390, η^2^*_G_* = 0.028, 95% CI, 0.00;1.00) and clarity on d’ (F(2.87, 258.72) = 349.918, *P* < .00001, η^2^*_G_* = 0.618, 95% CI, 0.00; 1.00). A two-way interaction between CAPS group and clarity, F(2.87, 258.72) = 3.026, *P* = .0320, η^2^*_G_* = 0.014, 95% CI, 0.00;1.00, showed that low-CAPS individuals were better able than high-CAPS individuals to distinguish regular and inverted vocoded words, particularly for high-clarity stimuli. The simple effect of CAPS group at single clarity levels was reliable at 100% clarity (F(1, 90) = 6.90, *P* = .01, η^2^*_G_* = 0.071, 95% CI, 0.01;1.00) and 95% clarity (F(1,90) = 4.27, *P* = .042, η^2^*_G_* = 0.045, 95% CI, 0.00;1.00). Pairwise comparisons show that the mean d’ was significantly different at 100% clarity (high-CAPS M = 1.57, low-CAPS M = 1.88, t(90) = -2.63, *P* = .0102, d = -0.588, 95% CI, -0.550;-0.0763), and 95% clarity (high-CAPS M = 1.01, low-CAPS M = 1.20, t(90) = -2.07, *P* = .0416, d = -0.495, 95% CI, -0.380;-0.00757).

A mixed ANCOVA including age as a covariate confirmed that CAPS group differences in sensitivity remained significant – CAPS: F(1,89) = 4.77, *P* = .032, η^2^*_G_* = 0.04, 95% CI, 0.00; 1.00; CAPS-by-clarity: F(2.93260.94) = 3.25, *P* = .023, η^2^*_G_* = 0.001, 95% CI, 0.00;1.00. Sensitivity increased with clarity – F(2.93260.94) = 39.71, *P* < .001, η^2^*_G_* = 0.67, 95% CI, 0.61;1.00. Age showed a trend-level effect - F(1,89) = 3.47, *P* = .066, η^2^*_G_* = 0.30, 95% CI, 0.00;1.00, and including age did not change the pattern of CAPS effects.

In brief, (1) as expected, increasing clarity was associated with an increasing capacity to identify speech; (2) individuals with low CAPS scores were generally better at accurately making this distinction, and (3) the difference in discrimination ability between low- and high-CAPS scorers was greater as clarity increased.

#### Bias toward Reporting that Speech Was Present or Absent (Criterion)

A two-way mixed ANOVA showed reliable effects of CAPS group and clarity level on criterion; with a significant main effect of CAPS group (F(1, 90) = 3.977, *P* < .05, η^2^*_G_* = 0.0360, 95% CI, 0.00;1.00), and a higher overall criterion – a bias toward reporting that speech was not present – in the low-CAPS group (M = 0.925), compared to the high-CAPS group (M = 0.687, t(454.79) = -2.92, *P* = .00372, d = -0.278, 95% CI, -0.396;-0.0776). There was also a significant main effect of clarity on criterion (F(2.15, 193.88) = 616.376, *P* < .05, η^2^*_G_* = 0.505, 95% CI, 0.45;1.00). The interaction between CAPS group and clarity was not significant (F(2.15, 193.88) = .040, *P* = .968, η^2^*_G_* = 0.000067, 95% CI, 0.00;1.00). In brief, there was a general bias to perceiving stimuli as non-speech, and this was reduced with increasing clarity. Moreover, the tendency was more marked in low-CAPS individuals, though this group difference was unaffected by clarity level.

One-sample t-tests indicated that criterion was significantly greater than 0 for both groups at all clarity levels, except at 100% clarity, where the mean criterion of the low-CAPS group was not significantly different from 0 (M = 0.0202, t(46) = 0.300, *P* = .766, d = 0.0437, 95% CI, -0.115;0.156), and the mean criterion of the high-CAPS group was significantly less than 0 (M = -0.223, t(45) = -2.472, *p* = .0174, d = -0.369, 95% CI, -0.405;-0.0412). These results indicate that both groups displayed a bias to say that speech was not present at 60%, 80%, 90%, and 95% clarity. At 100% clarity, the responses of low-CAPS individuals were unbiased, whereas high-CAPS individuals were biased toward reporting that speech was present.

Repeating the analyses with delusion-proneness (PDI) in place of CAPS revealed no significant main effects or interactions with clarity for sensitivity or criterion (all *P* > .12), suggesting that the perceptual effects were specific to hallucination-proneness.

In brief, this study demonstrated the expected effects of inversion on perception of speech, with regular but not inverted words being identified as potentially intelligible. Furthermore, manipulations of clarity influenced listeners’ ability to discriminate regular from inverted words and their bias to identify sounds as containing intelligible speech. Individual differences in hallucination-proneness were linked to these perceptual outcomes, such that individuals with low CAPS scores were better able to distinguish regular and inverted speech than those with high CAPS scores—especially at higher clarity levels. While participants generally showed a bias to saying that speech was absent—perhaps because of the requirement to report words when they judged that speech was present—this effect was attenuated with increasing clarity such that, at 100% clarity, no overall bias was discernible for individuals with low CAPS scores. However, individual differences in this bias were also observed—high-CAPS showed a reduced bias toward reporting “no speech” at low clarity and a bias toward reporting speech at the highest clarity level.

## Experiment 2

### Methods

#### Participants

One hundred (50 female) participants were recruited from Prolific Academic. Two participants were excluded due to incomplete data. Ninety-eight participants (48 female) were included in final analyses (age M(SD) = 43.55, (12.41), range 20-72 years). All were native monolingual English speakers and had no hearing impairment, literacy difficulties, or language-related disorders based on self-report.

Participants were paid £6/hour. Informed consent was obtained via Qualtrics, under a process approved by the Cambridge Psychology Research Ethics Committee. Participants completed the CAPS and the PDI before the experimental task (see Experiment 1, Questionnaires).

#### Stimuli

Eighty highly frequent and confusable monosyllabic English words were chosen. The words were synthesized and vocoded as in Experiment 1. Only regular vocoded words (not inverted) were used in this experiment.

#### Task Procedure

**Figure 3:**
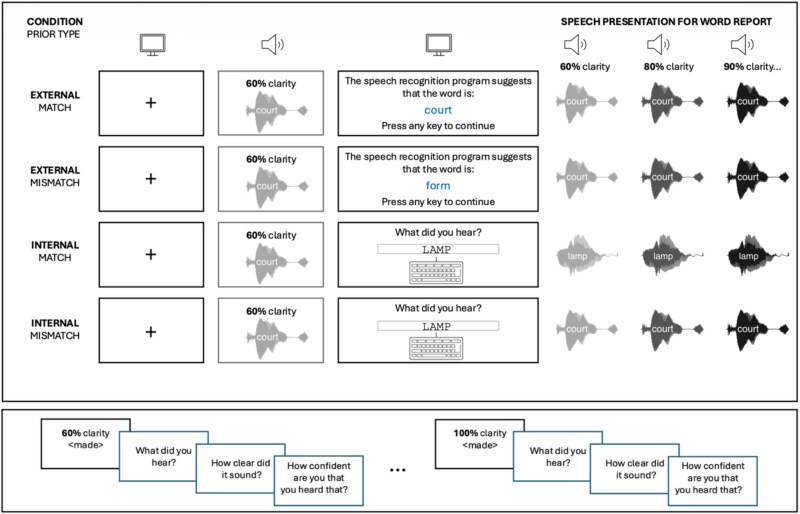
Top panel: Each trial starts with the “prior stage”—in the 2 external conditions, participants are shown a word on the computer screen, which either matches or mismatches the stimulus played at the prior generation stage, and the subsequently presented stimuli. In internal-matching condition, instead of seeing a word on the screen, participants are asked to generate a guess based on the stimulus they hear. This generated guess is then used as the auditory stimulus and played back to the participant at 5 increasing clarity levels. In internal-mismatching condition, participants are asked to generate a guess without seeing a displayed cue, but are then played the same stimulus they heard just before generating their guess.

The task consisted of 80 trials. As in Experiment 1, each trial consisted of 5 presentations of a vocoded word at 60%, 80%, 90%, 95%, and 100% clarity. This was preceded by an additional presentation of a vocoded stimulus at 60% clarity on each trial, which provided a “prior” for subsequent perception (Figure 3).

In the prior stage, alongside the auditory stimulus, participants were either shown a written word on the screen (providing an “external” prior for the degraded speech) or asked to type what word they heard (generating an “internal” prior based on their perception of degraded speech).

In the external prior condition, the written word either matched (ie, it was the same word that was presented at the prior stage and at all 5 subsequent clarity levels) or mismatched (ie, a written word was presented that differed from the spoken word). Mismatching words were chosen so that, for each word, the LR between the spoken word and its mismatching cue) was approximately 0.37, matching the average accuracy of typed responses for 60% morphed stimuli in Experiment 1. Participants were informed that the written word was a guess generated by a speech recognition program, which was not always accurate.

In the internal condition, at the prior stage, the vocoded stimulus was played at 60% clarity with no additional written cue on the screen. After participants heard the stimulus, they were asked to type what word they heard. Given that accuracy was relatively low and participants rarely guessed the word correctly at 60% clarity (5.71% of all cases), this initial response can be considered an internally generated prior for the perception of subsequently presented speech. To create an internal-match condition, subsequent presentations were manipulated to contain the word that participants reported (ie, the word participants typed at the prior stage was synthesized, vocoded, and played at the 5 increasing clarity levels). This was achieved by synthesizing and vocoding all monosyllabic words in the CELEX database, plus an additional 30 words that were not in the list but were generated by participants in pilot studies. The full list of 8055 words synthesized and vocoded can be found at: https://osf.io/3kpma/files/osfstorage. Participants were required to enter real English monosyllabic words, spelled correctly, to be able to proceed. The “continue” button was only enabled when the participant’s guess matched one of the words on this list.

In the internal-mismatch condition, there was no manipulation of the presented stimuli. Participants guessed the word correctly only in 5.71% of the cases, and hence in over 94% of trials their internal prior mismatched with the 5 subsequently presented stimuli. The 5.71% of trials where participants guessed the word correctly in the prior generation stage were excluded from further analyses.

For each participant, 20 words were randomly assigned to each of the 4 possible conditions: external-match, external-mismatch, and internal-match or internal-mismatch such that all words were encountered equally in all conditions. After each stimulus presentation (after the prior phase), they were asked to: (1) type what they heard, (2) rate the clarity of the stimulus (very unclear, unclear, moderately clear, clear, and very clear), and (3) give their confidence in their typed guess.

### Analysis

We report 2 outcome measures which describe participants’ perceptual performance in the task:

1. Report Accuracy: participants’ response accuracy, as measured by a LR measuring the similarity between the phonetic transcription of the typed responses and the stimulus (equivalent to the accuracy measure used in Experiment 1 (Supplement S1.1)).
2. Shift Index: the extent to which the words that participants reported were influenced (“shifted”) by internal/external priors and by the stimuli presented, and calculated as the response–stimulus minus response-prior LR. Negative values indicate a greater similarity to the prior; positive values indicate greater similarity to the stimulus, and a value of 0 means the guess is equally similar to both.

### Results

#### CAPS Scores

CAPS scores had a mean of 60.0 (SD = 60.4) and a median of 41. See Supplement S2.3 for distribution plots, correlations with PDI scores, and comparisons of CAPS score distributions across the 2 experiments.

### Overall Task Performance

To quantify overall perceptual performance in this task, we assessed how report accuracy, similarity to the prior, and shift index varied across the 4 combinations of prior condition (external and internal) and prior type (match and mismatch) for all participants.

### Report Accuracy

**Figure 4:**
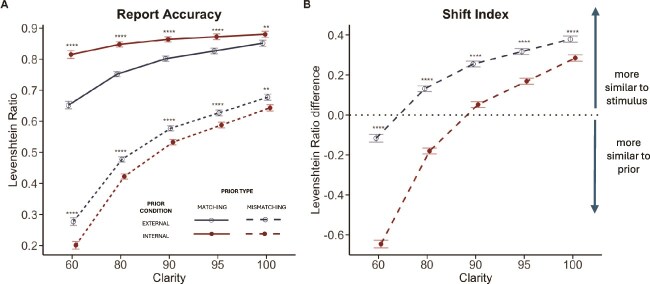
Task performance for all participants. (A) Estimated marginal means of the Levenshtein Ratio between participant’s word reports and the stimuli played (approximately equivalent to report accuracy), averaged across words and participants *(mean* ± SEM) at each clarity level. Asterisks indicate significant pairwise comparisons between external and internal conditions (^**^: *P* < .01, ^****^: *P* < .0001). (B) The difference between similarity to stimulus and similarity to prior for each word, averaged across all words and participants *(mean* ± SEM) at each clarity level for trials with mismatching priors. Negative values indicate perceptual reports that are more similar to the prior, and positive values indicate reports that are more similar to the stimulus played. The dotted line (shift index = 0) indicates that responses are equally similar to the prior and the stimulus. Bottom panel: Following the prior generation stage, the structure of the trial is the same for all 4 experimental conditions. Participants hear a word at 60%, 80%, 90%, 95%, and 100% clarity levels, and at each clarity level, they are asked to type what they hear, rate how clear the stimulus sounds to them, and rate how confident they are in their typed guess.

As expected, word report accuracy depended on prior condition, prior type, and stimulus clarity (Figure 4A). Participants were more accurate when they had a matching prior and when speech clarity was higher. Effects of prior condition interacted with prior type—internal priors led to increased report accuracy when priors matched the stimulus, but decreased accuracy for priors that mismatched. However, effects of prior knowledge on report accuracy were reduced at high clarity, as were differences between internal and external prior conditions. We provide detailed statistical analysis of these findings in supporting information (Supporting Materials S2.1.1).

### Shift Index

To quantify the influence of prior knowledge and sensory input on participants’ responses, we computed a shift index expressing how—in mismatching prior conditions—participants change from reporting the prior to reporting the stimulus. Only mismatching trials were included, as the shift index is always 0 in matching prior conditions where the prior and stimulus are identical.

The average shift index depended on prior condition (internal/external) and stimulus clarity (Figure 4B). Overall, participants’ responses were more similar to the prior than to the stimulus played. The shift index was lower in the internal prior than the external prior condition, and increased with increasing clarity in both conditions. Effects of prior condition interacted with clarity such that as clarity increased, the shift-index differences between internal and external conditions decreased. Statistical analyses of these findings are provided in Supporting Materials S2.1.3.

To summarize, we observed that report accuracy for degraded speech increased with increasing clarity for both the internal and external mismatch conditions. The internal mismatch condition led to initial responses that were more similar to the prior than the external-mismatch condition (ie, internally-generated priors more strongly influenced responses). As clarity increased, participants abandoned their mismatching priors and gave reports that were more similar to the stimulus (shown by positive shift-index values). However, for the external-mismatch condition, this change occurred at lower levels of clarity, showing that internally-generated priors continued to have an increased influence on participants’ responses even as clarity improved, and demonstrated that these mismatching priors were wrong. We now turn to the question of whether the effect of these priors on word identification differs according to hallucination-proneness.

### Effects of Hallucination-Proneness on Perceptual Performance

To investigate if hallucination-proneness influenced report accuracy and shift index in mismatching conditions, we added a fixed effect of CAPS score to the base models (described in Supporting Materials S2.1.1 for report accuracy and S2.1.3 for the shift index), and explored interactions with prior condition and prior type. Models were specified a priori to test CAPS-by-condition effects; CAPS-by-condition-by-clarity interactions were examined exploratorily, with model comparison statistics reported in the Supplement (S2.2.1). Satterthwaite approximations were used to estimate degrees of freedom for all t and F statistics.

### Report Accuracy

To test whether the effect of prior type (matching/mismatching) and prior condition (internal/external) on word report accuracy varies with CAPS scores, we added a fixed effect of CAPS score to the models fitted previously. The resulting model was:

average similarity to stimulus ~ clarity* prior condition * prior type + CAPS score * prior condition * prior type + (1|participant).

**Figure 5:**
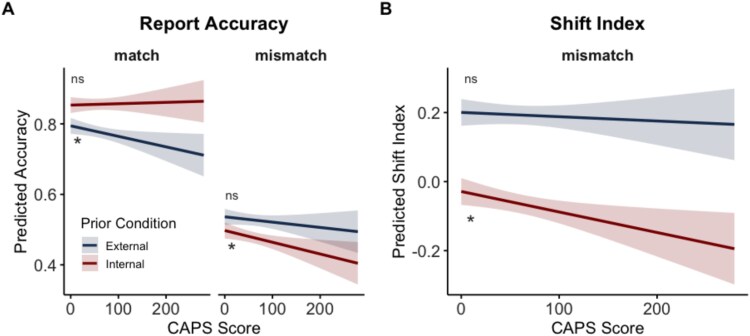
Estimated slopes of CAPS score. (A) Word report accuracy. Cardiff Anomalous Perceptions Scale score slopes were significantly steeper (more negative) in the external-match compared to the internal-match condition. Asterisks indicate significant slopes of CAPS score within each condition (*: *P* < .05, ns: *P* > .05). Cardiff Anomalous Perceptions Scale slopes were not significantly different between internal-mismatch and external-mismatch conditions, as indicated by overlapping confidence intervals. (B) The shift index was significantly more negative in internal-mismatch than external-mismatch condition.

Higher CAPS scores were associated with lower report accuracy overall (β = -0.000299, SE = 0.000135, 95% CI, -0.000563;-0.0000341, t = -2.21, *P* = .028). There was a significant interaction between prior condition and CAPS score (β = 0.000337, SE = 0.000121, 95% CI, 0.000101;0.000573, t = 2.79, *P* < .01), and a three-way interaction between prior condition, prior type, and CAPS score, F(1,1852) = 9.26, *P* < .01, β = -0.00052, SE = 0.000171, 95% CI, -0.000854;-0.000186, t = -3.04. These effects are summarized in Figure 5A which shows that reduced accuracy for participants with higher CAPS scores was most apparent for external match trials and for internal mismatch trials. To better characterize these effects, we obtained estimated slopes of CAPS score for each prior condition (external/internal) for each prior type (Figure 5A). To directly test whether the slope of CAPS score effects differed between internal and external prior conditions, we conducted pairwise comparisons of the estimated slopes in single conditions using estimated marginal trends.

For matching priors, the slope of CAPS score was positive but non-significant (β = 0.0000388, SE = 0.000135, 95% CI, -0.000228;0.000306, t(193) = 0.28691, *P* = .77) in the internal condition, and significantly negative in the external condition (β = -0.000299 SE = 0.000135, 95% CI, -0.000565;-0.0000316, t(193) = -2.21, *P* < .05). Moreover, the difference in the slopes between external and internal conditions was significant, β = -0.000337, SE = 0.000121, 95% CI, -0.000574;-0.000101, t(1852) = -2.794, *P* = .0053, suggesting that the relationship between accuracy and CAPS score was significantly more negative in the external than the internal condition when the prior matched the subsequently presented stimuli. These comparisons, therefore, show that for trials with matching priors, higher CAPS scores are associated with poorer accuracy, specifically for trials in which a matching, correct cue is provided from an external source.

When the prior was mismatching, the slope of CAPS score was negative but non-significant in the external condition (β = -0.000151, SE = 0.000135, 95% CI, -0.000418;0.000116, t(193) = -1.11, *P* = .267), and significantly negative in the internal condition (β = -0.000333, SE = 0.000135, 95% CI, -0.000600;-0.0000660, t(193) = -2.460, *P* = .0148). Cardiff Anomalous Perceptions Scale score slopes did not significantly differ between external and internal conditions, β = 0.000182, SE = 0.000121, 95% CI, -0.0000545;0.000419, t(1852) = 1.51, *P* = .131, suggesting that although CAPS score was significantly associated with lower accuracy in the internal-mismatch condition, the difference in this association between external and internal mismatch conditions was not significant.

To further explore effects of mismatching priors on word report, we next assess the relationship between shift index measures and CAPS scores.

### Shift Index

To test the hypothesis that effects of prior knowledge on perception differed between the 2 prior conditions depending on CAPS score, we added a fixed effect of CAPS score to our statistical analysis of the shift index, which assesses the relative influence of prior knowledge and stimulus in mismatching trials as participants hear progressively clearer speech, which over-rules their prior knowledge. The final model was:

average shift index ~ clarity* prior condition *+* CAPS score* prior condition + (1|participant).

Model comparisons indicated that a model with a CAPS score-by-condition interaction (AIC = -618) was a better fit than a model without (AIC = -611), χ^2^(2) = 10.4, *P* < .01. Model results showed that there was no main effect of CAPS score, F(1, 96) = 2.75, *P* = .101, β = -0.000124, SE = 0.000233, 95% CI, -0.000581;0.000444. There were main effects of clarity, F(1,878) = 2435.34, *P* < .0001, β = 0.0124, SE = 0.000511, 95% CI, 0.0114; 0.0134, and condition, F(1,878) = 334.10, *P* < .0001, β = -1.153, SE = 0.0631, 95% CI, -1.278;-1.029.There was a significant CAPS score-by-prior-condition interaction, F(1,878) = 7.64, *P* = .00582, (β = -0.000470, SE = 0.000170, 95% CI, -0.000804,-0.000136; t(972) = -2.76, *P* = .006). Exploratory analyses replacing CAPS with PDI revealed a significant condition-by-PDI interaction (*P* = .0175), similarly indicating greater reliance on internally generated priors with increasing delusion-proneness.

To examine how hallucination-proneness modulates reliance on internally-generated and externally-provided priors, we conducted simple slopes analyses of CAPS score within each prior condition. In the external prior condition, the slope relating CAPS score to shift index was only weakly negative and non-significantly different from zero (β = -0.000124, SE = 0.000233, 95% CI, -0.000585;0.000335; t = -0.534, *P* = .594), indicating that there was no overall effect of CAPS score on the influence of externally-provided mismatching priors. However, in the internal prior condition, the slope estimate was negative and statistically significant (β = -0.000594 SE = 0.00023, 95% CI, -0.00106;-0.000134, t(127) = -2.552, *P* = .0119), showing that individuals with higher CAPS scores are more strongly influenced by incorrect priors that have been generated internally.

To test whether CAPS score slopes were different between internal and external prior conditions, we conducted pairwise comparisons of simple slopes using emtrends. The difference in the slopes was significant, confirming a stronger negative association between CAPS scores and the shift index in the internal condition, β = -0.00047, SE = 0.00017, 95% CI, 0.000136;0.000804, t(878) = -2.764, *P* < .0058.

Overall, these results from Experiment 2 demonstrate that mismatching priors differentially affect perceptual outcomes in more hallucination-prone individuals when priors are internally generated compared to when equivalent priors are externally provided.

## Discussion

We developed 2 novel tasks involving the perception of vocoded speech to explore how hallucination-proneness, as indexed by CAPS scores, influences speech perception under ambiguous conditions, and to examine the role of different types of prior information in interpreting noisy speech.

Experiment 1 showed that participants with higher hallucination-proneness were less able to discriminate between regular vocoded speech and inverted, unintelligible stimuli at higher clarity levels. All participants showed an overall bias toward reporting that speech was absent—perhaps due to the task requirement to report the word that they heard. However, this bias was attenuated in those with higher hallucination-proneness. Notably, at the highest clarity level, only the high-proneness group showed a bias toward indicating that words were present - they often reported hearing words in unintelligible spectrally-rotated words. These results align with previous research linking hallucinations with an increased tendency to detect speech where none exists,^15^^,^^32^ and, perhaps more generally, an overall bias to detect meaningful signals in noise.^44^^,^^45^

Experiment 2 examined whether individuals with higher hallucination-proneness rely more on internally-generated than externally-provided perceptual priors when interpreting degraded speech. Results showed that higher hallucination-proneness was associated with a stronger influence of internally-generated priors on perceptual report. When these priors did not match the subsequent stimuli, more hallucination-prone individuals were more reluctant to discard incorrect priors in response to degraded stimuli. Conversely, when the prior did match the stimulus, higher hallucination-proneness was linked to poorer perceptual identification when the prior was externally provided, but not when it was internally generated. This suggests a further difficulty effectively using accurate external priors in high-proneness individuals.

These results support predictive processing accounts of hallucinations and show that at higher levels of the perceptual hierarchy, participants with higher hallucination-proneness demonstrate a stronger reliance on internally-generated priors when interpreting ambiguous auditory stimuli. This effect did not extend to externally-provided priors, despite both internal and external cues being equally informative in our tasks. Indeed, externally-provided priors were treated as less reliable by more hallucination-prone individuals when they matched an ambiguous stimulus. This asymmetry points to a source-specific difference in how prior information is weighted during perceptual inference. Our results suggest that internally-generated priors may also be treated as more reliable, thereby exerting disproportionate influence over perception (see^24^ for further discussion on source monitoring and predictive processing).

This interpretation is consistent with hierarchical predictive coding models of psychosis, which propose that a disruption in the process of estimating the precision of both predictions and prediction errors underlies symptoms in psychosis,^3^^,^^4^ with excessive precision attributed to high-level, internally-generated priors, leading to aberrant inferences about ambiguous stimuli. This would explain both the stronger influence of internal priors in our task and the reduced benefit from accurate, externally-provided cues. It may also offer a resolution to previously-reported, seemingly contrasting, findings that externally-directed expectations did not differentially modulate auditory perception in more hallucination-prone individuals.^15^ Our results suggest that the locus of these effects may not simply reflect the presence or absence of perceptual expectations, but that the source and perceived precision of perceptual predictions are also critical. Our data suggest that hallucination-proneness may be linked to 2 changes in the use of prior knowledge: (1) an increase in the precision of internally-generated predictions, which disrupts the balance of inference in auditory perception, and (2) a decrease in the precision of externally-generated predictions with a tendency to overlook correct expectations or perceptual interpretations provided by others, which suggests a mechanism for the maintenance of hallucinations.

We note that the prior in our internal condition is not categorically distinct from priors in other paradigms, as it reflects an internal representation formed from prior sensory input. The critical distinction in our design is whether this internally maintained belief is additionally shaped by highly precise externally-provided information.

The stronger reliance on internally-generated priors observed in our experiment may appear to contradict studies that demonstrated that individuals with higher hallucination-proneness benefit more than those with lower hallucination-proneness from externally-provided visual priors when interpreting ambiguous images.^8^ However, in contrast to our design, the priors in Teufel and colleagues^8^ could be considered as strongly shaped by externally provided information. Thus, the enhanced use of these templates by hallucination-prone individuals may reflect a more general tendency to rely on prior knowledge when sensory input is ambiguous, and an increased responsiveness to contextually relevant priors when their reliability is clear and high. In contrast, our findings suggest that when prior reliability is unclear, and priors are internally generated, participants with high hallucination-proneness show a disproportionate reliance on internal priors, even when they are incorrect, compared to externally-provided priors of identical informational value.

Hallucination-proneness may thus involve differential precision weighting of internally-generated and externally-provided priors. This could also account for the reduced benefit from veridical external priors in individuals with high hallucination-proneness. From a Bayesian standpoint, when a prior matches the incoming sensory input, it should enhance perceptual accuracy by optimizing the posterior belief. However, if externally-provided predictions are assigned insufficient precision, even accurate priors may fail to exert appropriate influence on perception. Internally generated priors may then persist in their influence, regardless of their accuracy, leading to perceptual experiences that are increasingly shaped by endogenous expectations rather than sensory inputs from the environment—a core feature of hallucinations.

This imbalance may disrupt belief updating by biasing Bayesian model selection in favor of internal models, even when unsupported by sensory evidence.

As a result, perceptual hypotheses driven by internally generated predictions may override contradictory sensory input, causing perceptual inflexibility and resistance to external correction. This aligns with proposals that hallucinations arise not only from overly precise internal predictions^1^^,^^6^^,^^13^^,^^46^^,^^47^ but also from a failure to suppress internally generated percepts^48-50^—overweighted internal priors may make even minor sensory mismatches highly surprising, prompting inference of an external cause and resulting in experiences that feel externally generated despite being internally produced.

Several limitations should be noted. We did not collect separate measures of depression, anxiety, or cognitive ability to reduce participant burden. Although screening reduced the likelihood of clinical comorbidity and the key sensitivity effects remained robust after adjusting for age, we cannot fully exclude shared variance with subclinical psychopathology or cognitive factors. Future work should incorporate standardized assessments of mood, anxiety, and cognition to tests specificity more directly. Hardware checks were also not included, although the robust clarity effects suggest that stimuli were perceived as intended. Future studies could incorporate standardized headphone-check procedures.^51^^,^^52^

Together, these results suggest that hallucination-proneness is characterized by stronger weighting of internally-generated predictions relative to externally provided ones during online perceptual inference, consistent with altered precision estimates for internally sourced signals. This imbalance may compromise the ability to learn from the environment and flexibly update beliefs, contributing to the persistence of internally-generated, inaccurate percepts characteristic of hallucinatory experiences.

Future work could test whether this internal/external asymmetry arises from differences in perceived credibility, a general distrust of external information, or reduced learning about external cue reliability. This could be done by manipulating beliefs about the accuracy of external cues, or by providing trial-by-trial feedback to encourage learning about cue validity, and examining whether people with higher hallucination-proneness show reduced learning or slower updating of their expectations.

## Supplementary Material

sbag048_Supporting_Materials_revised_3Jan2026_clean

## References

[1] Corlett PR, Horga G, Fletcher PC, Alderson-Day B, Schmack K, Powers AR III. Hallucinations and strong priors. *Trends Cogn Sci*. 2019;23:114-127. 10.1016/j.tics.2018.12.001 30583945 PMC6368358

[2] Powers AR, Mathys C, Corlett PR. Pavlovian conditioning–induced hallucinations result from overweighting of perceptual priors. *Science.* 2017;357:596-600. 10.1126/science.aan3458 28798131 PMC5802347

[3] Sterzer P, Adams RA, Fletcher P, et al. The predictive coding account of psychosis. *Biol Psychiatry*. 2018;84:634-643. 10.1016/j.biopsych.2018.05.015 30007575 PMC6169400

[4] Adams RA, Stephan KE, Brown HR, Frith CD, Friston KJ. The computational anatomy of psychosis. *Frontiers in psychiatry*. 2013;4:47. 10.3389/fpsyt.2013.00047 23750138 PMC3667557

[5] Kafadar E, Mittal VA, Strauss GP, et al. Modeling perception and behavior in individuals at clinical high risk for psychosis: support for the predictive processing framework. *Schizophr Res*. 2020;226:167-175. 10.1016/j.schres.2020.04.017 32593735 PMC7774587

[6] Powers AR III, Kelley M, Corlett PR. Hallucinations as top-down effects on perception. *Biol Psychiatry: Cognit Neurosci Neuroimaging*. 2016;1:393-400. 28626813 10.1016/j.bpsc.2016.04.003 PMC5469545

[7] Kafadar E, Fisher VL, Quagan B, et al. Conditioned hallucinations and prior overweighting are state-sensitive markers of hallucination susceptibility. *Biol Psychiatry*. 2022;92:772-780. 10.1016/j.biopsych.2022.05.007 35843743 PMC10575690

[8] Teufel C, Subramaniam N, Dobler V, et al. Shift toward prior knowledge confers a perceptual advantage in early psychosis and psychosis-prone healthy individuals. *Proc Natl Acad Sci*. 2015;112:13401-13406. 10.1073/pnas.1503916112 26460044 PMC4629373

[9] Davies DJ, Teufel C, Fletcher PC. Anomalous perceptions and beliefs are associated with shifts toward different types of prior knowledge in perceptual inference. *Schizophr Bull*. 2018;44:1245-1253. 10.1093/schbul/sbx177 29294128 PMC6192467

[10] Zarkali A, Adams RA, Psarras S, Leyland L-A, Rees G, Weil RS. Increased weighting on prior knowledge in Lewy body-associated visual hallucinations. *Brain communications*. 2019;1:fcz007. 10.1093/braincomms/fcz007 PMC6924538 31886459

[11] Stuke H, Kress E, Weilnhammer VA, Sterzer P, Schmack K. Overly strong priors for socially meaningful visual signals are linked to psychosis proneness in healthy individuals. *Front Psychol*. 2021;12:583637. 10.3389/fpsyg.2021.583637 33897518 PMC8061414

[12] Kot T, Serper M. Increased susceptibility to auditory conditioning in hallucinating schizophrenic patients: a preliminary investigation. *J Nerv Ment Dis*. 2002;190:282-288. 10.1097/00005053-200205000-00002 12011606

[13] Benrimoh D, Fisher VL, Seabury R, et al. Evidence for reduced sensory precision and increased reliance on priors in hallucination-prone individuals in a general population sample. *Schizophr Bull*. 2024;50:349-362. 10.1093/schbul/sbad136 37830405 PMC10919780

[14] Alderson-Day B, Lima CF, Evans S, et al. Distinct processing of ambiguous speech in people with non-clinical auditory verbal hallucinations. *Brain.* 2017;140:2475-2489. 10.1093/brain/awx206 29050393

[15] Alderson-Day B, Moffatt J, Lima CF, et al. Susceptibility to auditory hallucinations is associated with spontaneous but not directed modulation of top-down expectations for speech. *Neuroscience of consciousness*. 2022;2022:niac002. 10.1093/nc/niac002 PMC8824703 35145758

[16] Knolle F, Sterner EF, Demler VF, MacGregor LJ, Mathys C. Guided by expectations: overweighted semantic priors in schizotypy and their links to glutamate. *Biol Psychiatry*. 2026;99:569‐579. 10.1016/j.biopsych.2025.06.025 40623619

[17] Davis MH, Johnsrude IS. Hierarchical processing in spoken language comprehension. *J Neurosci*. 2003;23:3423-3431. 10.1523/jneurosci.23-08-03423.2003 12716950 PMC6742313

[18] Belin P, Fecteau S, Bedard C. Thinking the voice: neural correlates of voice perception. *Trends Cogn Sci*. 2004;8:129-135. 10.1016/j.tics.2004.01.008 15301753

[19] Hickok G, Poeppel D. The cortical organization of speech processing. *Nat Rev Neurosci*. 2007;8:393-402. 10.1038/nrn2113 17431404

[20] Friston KJ, Sajid N, Quiroga-Martinez DR, Parr T, Price CJ, Holmes E. Active listening. *Hear Res*. 2021;399:107998. 10.1016/j.heares.2020.107998 32732017 PMC7812378

[21] Davis MH, Sohoglu E. Three functions of prediction error for Bayesian inference in speech perception. *The cognitive neurosciences*. Cambridge (MA): MIT Press; 2020. p. 177–89. 10.7551/mitpress/11442.003.0022

[22] Heilbron M, Armeni K, Schoffelen J-M, Hagoort P, De Lange FP. A hierarchy of linguistic predictions during natural language comprehension. *Proc Natl Acad Sci*. 2022;119:e2201968119. 10.1073/pnas.2201968119 35921434 PMC9371745

[23] Fletcher PC, Frith CD. Perceiving is believing: a Bayesian approach to explaining the positive symptoms of schizophrenia. *Nat Rev Neurosci*. 2009;10:48-58. 10.1038/nrn2536 19050712

[24] Griffin JD, Fletcher PC. Predictive processing, source monitoring, and psychosis. *Annu Rev Clin Psychol*. 2017;13:265-289. 10.1146/annurev-clinpsy-032816-045145 28375719 PMC5424073

[25] Vinogradov S, Willis-Shore J, Poole JH, Marten E, Ober BA, Shenaut GK. Clinical and neurocognitive aspects of source monitoring errors in schizophrenia. *Am J Psychiatry*. 1997;154:1530-1537. 10.1176/ajp.154.11.1530 9356560

[26] Brookwell M, Bentall R, Varese F. Externalizing biases and hallucinations in source-monitoring, self-monitoring and signal detection studies: a meta-analytic review. *Psychol Med*. 2013;43:2465-2475. 10.1017/S0033291712002760 23282942

[27] Garrison JR, Fernandez-Egea E, Zaman R, Agius M, Simons JS. Reality monitoring impairment in schizophrenia reflects specific prefrontal cortex dysfunction. *Neuroimage Clin*. 2017;14:260-268. 10.1016/j.nicl.2017.01.028 28203529 PMC5292760

[28] Simons JS, Davis SW, Gilbert SJ, Frith CD, Burgess PW. Discriminating imagined from perceived information engages brain areas implicated in schizophrenia. *Neuroimage.* 2006;32:696-703. 10.1016/j.neuroimage.2006.04.209 16797186

[29] Keefe RS, Arnold M, Bayen U, Harvey P. Source monitoring deficits in patients with schizophrenia; a multinomial modelling analysis. *Psychol Med*. 1999;29:903-914. 10.1017/S0033291799008673 10473317

[30] TS Woodward, M Menon. Misattribution models (II): source monitoring in hallucinating schizophrenia subjects. In: Jardri R, Cachia A, Thomas P, Pins D, editors. The Neuroscience of Hallucinations. New York (NY): Springer; 2012:169-184, 10.1007/978-1-4614-4121-2_10

[31] Damiani S, Donadeo A, Bassetti N, et al. Understanding source monitoring subtypes and their relation to psychosis: a systematic review and meta-analysis. *Psychiatry Clin Neurosci*. 2022;76:162-171. 10.1111/pcn.13338 35124869 PMC9313862

[32] Erb J, Kreitewolf J, Pinheiro AP, Obleser J. Aberrant perceptual judgments on speech-relevant acoustic features in hallucination-prone individuals. *Schizophr Bull Open*. 2020;1:sgaa059. 10.1093/schizbullopen/sgaa059

[33] Baayen RH, Piepenbrock R, Gulikers L. The CELEX Lexical Database (cd-Rom). Philadelphia (PA): Linguistic Data Consortium; 1996.

[34] Zoefel B, Allard I, Anil M, Davis MH. Perception of rhythmic speech is modulated by focal bilateral transcranial alternating current stimulation. *J Cogn Neurosci*. 2020;32:226-240. 10.1162/jocn_a_01490 31659922 PMC7212037

[35] Aller M, Økland HS, MacGregor LJ, Blank H, Davis MH. Differential auditory and visual phase-locking are observed during audio-visual benefit and silent lip-reading for speech perception. *J Neurosci*. 2022;42:6108-6120. 10.1523/JNEUROSCI.2476-21.2022 35760528 PMC9351641

[36] Scott SK, Blank CC, Rosen S, Wise RJ. Identification of a pathway for intelligible speech in the left temporal lobe. *Brain.* 2000;123:2400-2406. 10.1093/brain/123.12.2400 11099443 PMC5630088

[37] De Leeuw JR, Gilbert RA, Luchterhandt B. jsPsych: enabling an open-source collaborative ecosystem of behavioral experiments. *Journal of Open Source Software*. 2023;8:5351. 10.21105/joss.05351

[38] Lange K, Kühn S, Filevich E. “Just another tool for online studies”(JATOS): an easy solution for setup and management of web servers supporting online studies. *PLoS One*. 2015;10:e0130834. 10.1371/journal.pone.0130834 26114751 PMC4482716

[39] Bell V, Halligan PW, Ellis HD. The Cardiff anomalous perceptions scale (CAPS): a new validated measure of anomalous perceptual experience. *Schizophr Bull*. 2006;32:366-377. 10.1093/schbul/sbj014 16237200 PMC2632213

[40] Peters E, Joseph S, Day S, Garety P. Measuring delusional ideation: the 21-item Peters delusions inventory (PDI). *Schizophr Bull*. 2004;30:1005-1022. 10.1093/oxfordjournals.schbul.a007116 15954204

[41] Macmillan NA . Signal detection theory. *Stevens’ handbook of experimental psychology: Methodology in experimental psychology*. 2002;3:43-90. 10.1002/0471214426.pas0402

[42] Sohoglu E, Davis MH. Perceptual learning of degraded speech by minimizing prediction error. *Proc Natl Acad Sci*. 2016;113:E1747-E1756. 10.1073/pnas.1523266113 26957596 PMC4812728

[43] Levenshtein V . Binary codes capable of correcting deletions, insertions, and reversals. Sov Phys Dokl. 1966;10:845‐848.

[44] Grant P, Balser M, Munk AJL, Linder J, Hennig J. A false-positive detection bias as a function of state and trait schizotypy in interaction with intelligence. *Frontiers in psychiatry*. 2014;5:135. 10.3389/fpsyt.2014.00135 25309464 PMC4174737

[45] Kapur S . Psychosis as a state of aberrant salience: a framework linking biology, phenomenology, and pharmacology in schizophrenia. *Am J Psychiatry*. 2003;160:13-23. 10.1176/appi.ajp.160.1.13 12505794

[46] Horga G, Abi-Dargham A. An integrative framework for perceptual disturbances in psychosis. *Nat Rev Neurosci*. 2019;20:763-778. 10.1038/s41583-019-0234-1 31712782

[47] Benrimoh D, Parr T, Vincent P, Adams RA, Friston K. Active inference and auditory hallucinations. *Computational Psychiatry (Cambridge, Mass)*. 2018;2:183. 10.1162/CPSY_a_00022 30627670 PMC6317754

[48] CD Frith . The Cognitive Neuropsychology of Schizophrenia: Classic edition. Hove (UK): Psychology press; 2015, 10.4324/9781315785011.

[49] Bentall RP . The illusion of reality: a review and integration of psychological research on hallucinations. *Psychol Bull*. 1990;107:82-95. 10.1037/0033-2909.107.1.82 2404293

[50] Feinberg I . Efference copy and corollary discharge: implications for thinking and its disorders. *Schizophr Bull*. 1978;4:636-640. 10.1093/schbul/4.4.636 734369

[51] Woods KJP, Siegel MH, Traer J, McDermott JH. Headphone screening to facilitate web-based auditory experiments. *Attention, Perception, & Psychophysics*. 2017;79:2064-2072. 10.3758/s13414-017-1361-2 PMC5693749 28695541

[52] Milne AE, Bianco R, Poole KC, et al. An online headphone screening test based on dichotic pitch. *Behav Res Methods*. 2021;53:1551-1562. 10.3758/s13428-020-01514-0 33300103 PMC7725427

