# Supplementary material for: Hallucination Proneness is Linked to Over-Reliance on Internal Priors for Noisy Speech
Source: Schizophr Bull. 2026 May 9;52(3):sbag048. doi: 10.1093/schbul/sbag048 (PMC13156500; doi:10.1093/schbul/sbag048)
Supplement: sbag048_Supporting_Materials_revised_3Jan2026_clean [file sbag048_supporting_materials_revised_3jan2026_clean.docx]

SUPPORTING MATERIALS

Table of Contents

[Section 1: Experiment 1 2](#_Toc218519461)

[S1.1 Overall Task Performance 2](#_Toc218519462)

[S1.2 Effects of hallucination proneness on perceptual performance 3](#_Toc218519463)

[S1.2.1 Model Comparisons 3](#_Toc218519464)

[S1.2.2 Word report accuracy 3](#_Toc218519465)

[S1.3 Distributions of CAPS and PDI scores 5](#_Toc218519466)

[Section 2: Experiment 2 5](#_Toc218519467)

[S2.1 Overall Task Performance 5](#_Toc218519468)

[S2.1.1 Similarity to Stimulus (Report Accuracy) 5](#_Toc218519469)

[S2.1.2 Similarity to Prior 6](#_Toc218519470)

[S2.1.3 Shift Index 8](#_Toc218519471)

[S2.2 Effects of hallucination proneness on perceptual performance 9](#_Toc218519472)

[S2.2.1 Model comparisons 9](#_Toc218519473)

[S2.2.1 Similarity to Prior 10](#_Toc218519474)

[S2.2.2 Shift Index 12](#_Toc218519475)

[S2.2.3 Additional visualisation of hallucination-proneness effects 12](#_Toc218519476)

[S2.3 Distribution of CAPS and PDI scores 13](#_Toc218519477)

[S2.4 Robustness checks 13](#_Toc218519478)

[Section 3: Stimuli 14](#_Toc218519479)

[S3.1 List of spoken words 14](#_Toc218519480)

# Section 1: Experiment 1

## S1.1 Overall Task Performance

A two-way repeated-measures ANOVA was performed to assess the effects of condition (regular and inverted) and clarity on the proportion of trials on which participants indicated that they heard a word (Figure 4a). There were main effects of condition, F(1,91)=589.242, p<0.05, and clarity, F(2.19, 198.90)=564.370, p<0.05, and a significant condition-by-clarity interaction, F(2.31, 209.76)=405.884, p<0.05 reflecting increased divergence of responses to regular and inverted stimuli at high clarity. Nonetheless, pairwise comparisons (paired t-tests) showed significant differences between regular and inverted trials at all clarity levels (Bonferroni-adjusted, all p<0.05) (Figure 4a).

A two-way repeated-measures ANOVA on the effects of condition and clarity on the proportion of fully correct word reports (Figure 4b) indicated a main effect of condition, F(1,45)=118.041, p<0.05 and clarity, F(2.55,114.79)=50.852, p<0.05, and a condition-clarity interaction, F(2.66, 119.68)=61.152, p<0.05.

Similarly, a two-way repeated-measures ANOVA was performed to examine the effects of clarity and condition (regular vs inverted words) on word report accuracy (Levenshtein Ratio, Figure 4c). There was a main effect of condition, F(1,45)=312.373, p<0.05, clarity, F(2.28, 102.66)=23.060, p<0.05, and a condition-clarity interaction, F(2.88, 129.79)=97.480, p<0.05.


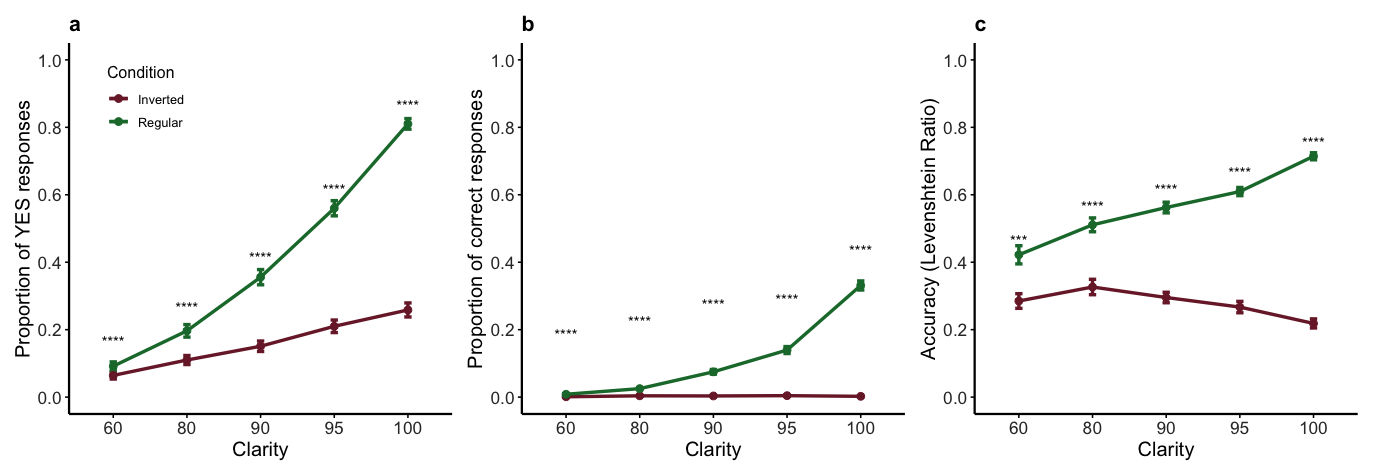
**FIGURE 1.** Task performance for all participants. a) Proportion of trials on which participants responded “yes” to “is there a word” for regular and inverted words (mean±SEM) at each clarity level. Asterisks indicate significant pairwise comparisons between the two conditions (***: p<0.001, ****: p<0.0001). b) Proportion of trials on which participants typed a fully correct response which matched the word being played after answering “yes” to “is there a word?” (mean±SEM) at each clarity level. c) Accuracy (Levenshtein Ratio) of typed responses (mean±SEM) at each clarity level.


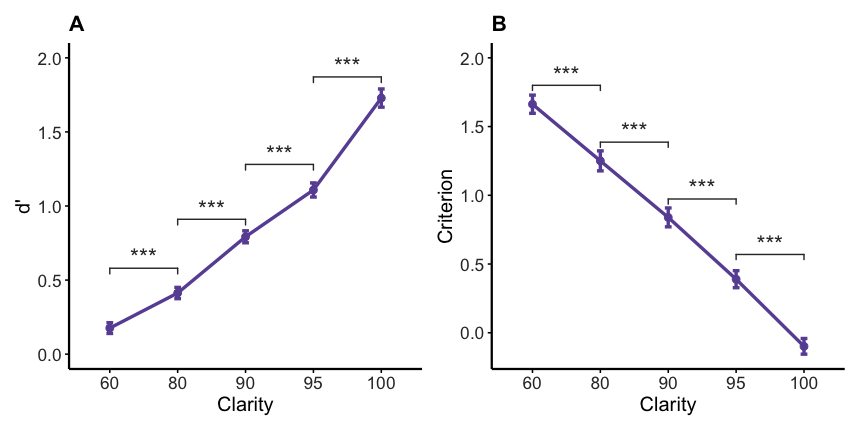


**FIGURE 2**. Task performance for all participants. A) d’ (sensitivity to distinguish regular and inverted words) and B) criterion values (mean±SEM, positive values indicate bias towards responding that words are not present) at each clarity level for all participants. Asterisks (***) indicate significant pairwise comparisons (p<0.001) between clarity levels.

## S1.2 Effects of hallucination proneness on perceptual performance

### S1.2.1 Model Comparisons

#### Continuous CAPS vs median-split

We investigated differences in perceptual performance between low-CAPS and high-CAPS individuals based on a median split over all participants. We also fitted linear models with CAPS score as a predictor, but these had higher AIC (e.g., 411.81 for d’) than models using the CAPS median-split to group participants into high- and low-CAPS (AIC=410.33 ) and did not significantly improve model fit compared to a model with clarity only (χ²(1) = 2.91, p=0.088), whereas models with CAPS group improved model fit (χ²(1) = 4.3941, p=0.036).

### S1.2.2 Word report accuracy

We report word report accuracy in regular (Figure 3a) and inverted (Figure 3b) trials on which participants indicated that they can hear a word in the sound played.

For words in the regular condition, there was a main effect of clarity level on the accuracy of typed responses (Levenshtein Ratio), F(1.97, 118.2) = 74.303, p<.0001 with more accurate responses at higher levels of clarity. The main effect of CAPS group on the accuracy of typed responses was not significant, F(1, 60)=2.417, p=0.125). A two-way ANOVA test indicated a statistically significant interaction between CAPS group and clarity level on the accuracy of typed responses (Levenshtein Ratio), F(1.97, 118.2)=3.413, p=.023. To understand the nature of this interaction we assessed the effect of CAPS group on word report accuracy at individual levels of clarity.

There was a simple effect of CAPS group at 60% clarity (F(1,60)=6.02, p=.017, Bonferroni-adjusted p=0.085). The simple effect of group was not significant at 80 (p=.778), 90 (p=.48), 95 (p=.502) and 100% (p=.412) clarity levels.

There was a statistically significant simple effect of clarity level on average LR for both high (F(1.82, 59.9)=57.2, p<0.001) and low CAPS (F(2.13, 57.6)=24.1, p<0.001) groups. Pairwise comparisons were significant between all clarity levels (p<0.05) except 80 and 90%, and 90 and 95% for high CAPS. For low CAPS, all pairwise comparisons were significant (p<0.05) except 60 and 80, 60 and 90, 80 and 90, and 90 and 95%. Due to violations of normality and the unequal numbers of observations at each clarity level (due to fewer word reports at lower clarity levels), we hesitate to draw strong conclusions from the interaction and post-hoc tests.

A mixed ANCOVA including age as a covariate indicated a small effect of age on accuracy (F(1,59)=4.51, p=.038). After adjusting for age, CAPS group effects were attenuated (F(1,59)=1.93, p=.17), and the CAPS x clarity interaction was trend-level (F(2.01,118.56)=2.99, p=.054), suggesting that age contributes modestly to variance in accuracy.


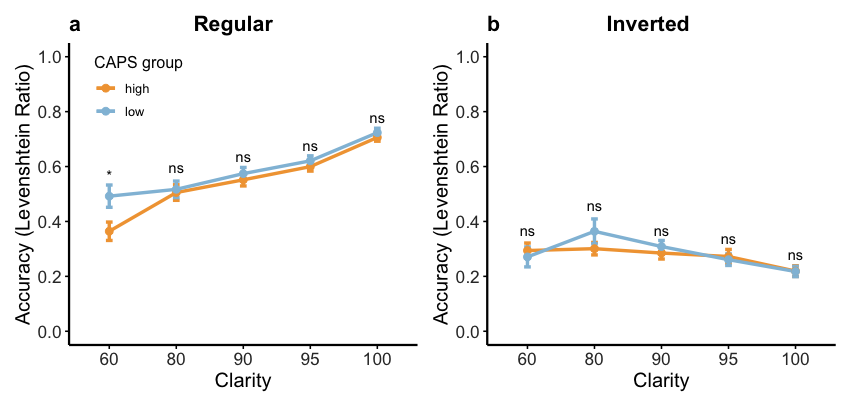
For inverted words, there was a main effect of clarity, F(2.96, 144.99)=6.402, p<0.05 with accuracy of word-report reducing at higher clarity levels. The main effect of CAPS group, F(1,49)=0.004, p=0.946 and the interaction between CAPS group and clarity, F(2.96, 144.99)=0.085, p=0.967, were not significant.

**FIGURE 3.** Average accuracy (Levenshtein ratio) of typed responses for high and low CAPS participants for regular (a) and inverted (b) words at each clarity level. Asterisks indicate significant pairwise comparisons between the two groups (*: p<0.05)

## S1.3 Distributions of CAPS and PDI scores

PDI scores had a mean of 31.2 (SD=29.7) and a median of 23.5. CAPS and PDI scores correlated (Pearson’s r=0.536, t=6.027, p<.01, Figure 4).


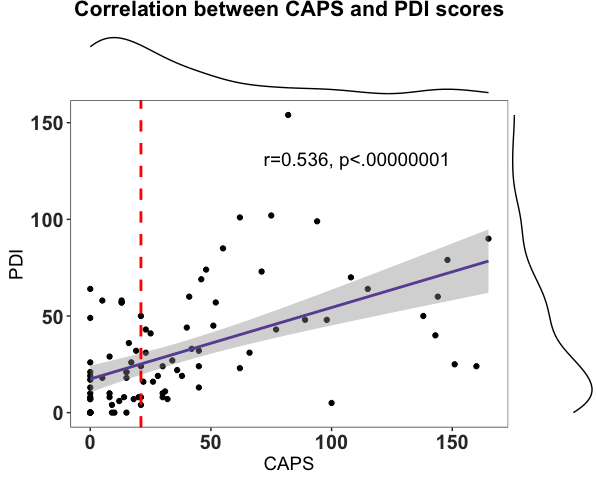


**FIGURE 4.** Proneness to hallucinations (CAPS) and delusions (PDI) were significantly correlated (N=92). The dashed vertical line shows median CAPS score used to split participants into low and high CAPS groups.

# Section 2: Experiment 2

## S2.1 Overall Task Performance

### S2.1.1 Similarity to Stimulus (Report Accuracy)

We used a linear mixed effects model to investigate how word report accuracy (average Levenshtein Ratio per participant at each clarity level) changed across the four different condition and type combinations. The model included fixed effects of condition, prior type, and clarity, with random intercepts for participants, specified as follows:

similarity to stimulus ~ condition * type * clarity + (1 | participant)

Model comparisons using likelihood ratio tests with a chi-squared distribution indicated that models including prior condition (χ²(2) = 18.337, p<0.001), prior type (χ²(2) = 1261.7, p<0.001), and clarity (χ²(2) = 407.98, p<0.001), provided a better fit than the null model.

F-tests using Satterthwaite’s method indicated significant main effects of clarity, F(1,1855)=1447.17, p<0.001, prior condition, F(1,1855)=12.90, p<0.001, and prior type, F(1,1855)=877.025, p<0.001. Model summary output showed that accuracy increased as clarity increased (β=0.00498, SE=0.000364, t(1950) = 13.70, p<0.001). Accuracy was higher in the internal condition compared to the external condition (β=0.14, SE=0.01, t(1938)=9.83, p<0.001), and lower when priors mismatched subsequently presented stimuli (β=-0.68, SE=0.04, t(1950)=-15.26, p<0.001).

There was a significant interaction between clarity and prior condition, F(1,1855)=10.04, p<0.01, β=-0.00336, SE=0.00051, t=-6.42, clarity and prior type, F(1,1855)=394.93, p<0.001, β=0.00502, SE=0.000514, t=9.77, and prior condition and prior type, F(1,1855)=64.50, p<0.001, β=-0.50, SE=0.06, t=-8.03. The three-way interaction between clarity, prior condition, and prior type was also significant, F(1,1855)=36.69, p<0.001, β=0.0044, SE=0.00073, p<0.001.

Pairwise comparisons using estimated marginal means showed that accuracy was lower in external-match compared to internal-match condition, β=-0.0791, SE=0.00727, t=-10.868, p<0.0001, but higher in external-mismatch compared to internal-mismatch, β=0.0498, SE=0.00727, t=6.849, p<0.0001. All other pairwise comparisons between the different condition and prior type combinations were also significant, p<0.001. All pairwise comparisons between internal and external conditions were significant at each clarity level in both matching and mismatching trials, p<0.01.

### S2.1.2 Similarity to Prior


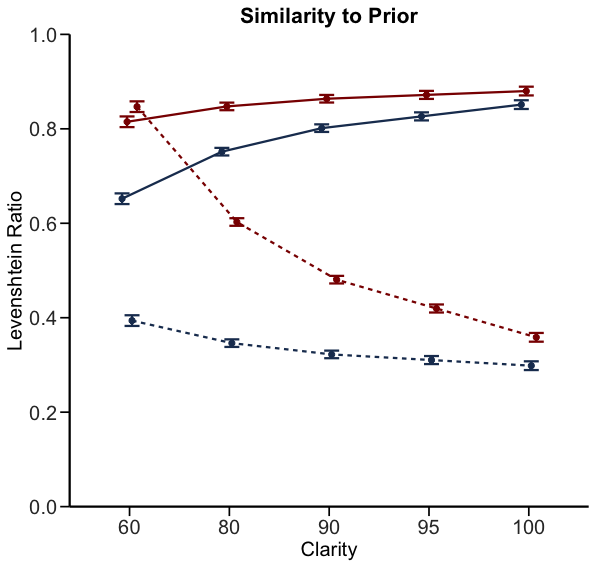

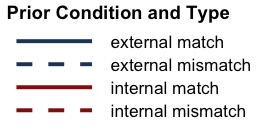


**FIGURE 5.** Estimated marginal means of the Levenshtein Ratio between participants’ word reports and priors at each clarity level (mean±SEM).

The average similarity between word report and prior also depended on prior condition, prior type, and stimulus clarity (Figure 5).Participants gave reports that were more similar to the prior where these matched the stimulus (matching priors). Following mismatching priors responses increasingly diverged from the prior as clarity increased with internal and external prior conditions showed similar effects of clarity and prior type. For low clarity stimuli (60%), however, participants gave equally similar responses for stimuli that matched or mismatched with internal priors whereas participants were more likely to give responses that resembled matching external priors than internal priors. Detailed statistics on these measures are reported in supporting information.

We used a linear mixed effects model to investigate how similarity of word reports to priors (difference in Levenshtein Ratios between word reports and priors, averaged across all words for each participant at each clarity level) changed across the four different condition and type combinations. The model included fixed effects of condition, prior type, and clarity, with random intercepts for participants, specified as follows:

similarity to prior ~ condition * type * clarity + (1 | participant)

Model comparisons using likelihood ratio tests with a chi-squared distribution indicated that models including condition (χ²(2) = 169.81, p<0.001), prior type (χ²(2) = 1655.8, p<0.001), and clarity (χ²(2) = 30.337, p<0.001), provided a better fit than the null model.

F-tests using Satterthwaite’s method indicated significant main effects of clarity, F(1,1855)=151.040, p<0.001, condition, F(1,1855)=630.609, p<0.001, and prior type, F(1,1855)=348.856, p<0.001.

Model summary output showed that similarity to prior increased as clarity increased (β=0.00498, SE=0.000325, t(1950) = 15.33, p<0.001). Similarity to prior was higher in the internal condition compared to the external condition (β=0.36, SE=0.04, t(1950)=9.20, p<0.001), and when priors mismatched subsequently presented stimuli (β=0.18, SE=0.04, t(1950)=4.65, p<0.001).

There was a significant interaction between clarity and condition, F(1,1855)=410.975, p<0.01, β=-0.00336, SE=0.00046, t=-7.30, clarity and type, F(1,1855)=1064.142, p<0.001, β=-0.00737, SE=0.00046, t=-16.03, and condition and type, F(1,1855)=146.560, p<0.001, β=0.68, SE=0.06, t=12.11. The three-way interaction between clarity, condition, and prior type was also significant, F(1,1855)=98.993, p<0.001, β=-0.00647, SE=0.00065, t=-9.95.

Pairwise comparisons using estimated marginal means showed that similarity to prior was lower in external compared to internal condition, β=-0.143, SE=0.0046, t(1855)=-31.162, p<0.0001, and higher in matching compared to mismatching prior type trials, β=0.378 SE=0.0046, t(1855)=82.263, p<0.0001. All pairwise comparisons between the different condition and prior type combinations were significant, p<0.001. All pairwise comparisons between internal and external conditions were significant at each clarity level in both matching and mismatching trials, p<0.01.

### S2.1.3 Shift Index

**Our fitted linear effects model** included fixed effects of condition and clarity, and random intercepts for participants:

average shift index ~ prior condition * clarity + (1 | participant)

Model comparisons using likelihood ratio tests with a chi-squared distribution indicated that models including condition (χ²(2) = 150, p<0.001), and clarity (χ²(2) = 775, p<0.001) provided a better fit than the null model. F-tests using Satterthwaite’s method indicated significant main effects of clarity, F(1,879)=2417, p<0.001, and condition, F(1,879)=357, p<0.001, and a significant clarity-by-condition interaction, F(1,879)=225, p<0.001.

Model summary output showed that the intercept was significantly negative, β=-0.86, SE=0.05, t(974)=-18.78, p<0.001, showing that participants’ responses were (at all clarity levels) more similar to the prior than to the stimulus played. Increasing clarity had a positive effect on the shift index, β=0.01, SE=0.00051, t(974)=24.16, p<0.001, and the internal prior condition was associated with a significantly lower shift index compared to the external condition, β=-1.18, SE=0.06, t(974)=-18.90, p<0.01. The significant clarity-by-condition interaction showed that as clarity increased, shift-index differences between internal and external priors were reduced. Pairwise comparisons showed that the internal-external difference was largest at 60% clarity, β=-0.529 SE=0.021, t(879)=-25.395, p<0.0001, and smallest at 100% clarity, β=-0.0941, SE=0.0150, t(879)=-6.295 p<0.0001.

A shift index of 0 indicates that the guess is equally similar to the prior and the stimulus played. We ran pairwise comparisons using estimated marginal means at each clarity level to see if shift index differed significantly from 0 in each condition. All comparisons were significant (Bonferroni-adjusted p<0.001). In the external condition, the estimated marginal mean was only significantly lower than 0 at 60% clarity, β=-0.12 SE=0.0191, t(378)=-6.128, p<0.0001. At all other clarity levels, the shift index was significantly greater than 0 (all p<0.001). In the internal condition, the estimated mean shift index was significantly negative at both 60% and 80% clarity, and positive at 90 (p=0.0008), 95 (p<0.0001) and 100% (p<0.0001) clarity levels. This difference further shows how internally-generated priors continued to have a stronger influence on participants’ reports of degraded speech at intermediate clarity levels.

## S2.2 Effects of hallucination proneness on perceptual performance

### S2.2.1 Model comparisons

#### Continuous CAPS score vs median-split

As in Experiment 1, we also fitted models with a high-/low-CAPS group factor as a predictor – based on a median split as before - but these models had higher AIC (e.g. -2751.0 for accuracy) than models with CAPS score as a linear predictor (AIC=-2757.2), and did not significantly improve model fit compared to the null model (χ²(4) = 8.4081, p=0.078 for accuracy, χ²(2) = 3.59, p=0.166 for the shift index). In contrast, models with CAPS score as a linear predictor showed improved model fit (χ²(4) = 14.4, p<0.01 for accuracy, χ²(2) = 10.408, p<0.01 for the shift index). We therefore used CAPS score as a continuous measure in the analyses that follow.

#### Condition-by-Type-by-CAPS vs Condition-by-Type-by-Clarity-by-CAPS

**REPORT ACCURACY**

To test whether the effect of prior type (matching/mismatching) and prior condition (internal/external) on word report accuracy varies with CAPS scores, we added a fixed effect of CAPS score to the models fitted previously. We compared this to a more complex model which also included an interaction with clarity. This more complex model did not provide a significantly better fit (AIC=-2511.995, BIC=-2411.542) than a model without CAPS score-by-clarity interaction terms (AIC=-2602.561, BIC=-2524.431). Furthermore, model comparisons using likelihood ratio tests indicated that the more complex model did not provide a better fit, χ²(4) = 6.27, p=0.18. Model comparisons indicated that a model with a CAPS score-by-condition-by-type interaction (AIC=-2757.2) provided a better fit for the data than a model without (AIC=-2750.6), χ²(4) = 14.54, p<0.01.

**SHIFT INDEX**

The more complex model did not provide a significantly better fit (AIC=-618, BIC=-569) than a model without a CAPS score-by-clarity interaction (AIC=-619, BIC=-579). This was supported by model comparisons using likelihood ratio tests which indicated that the more complex model did not provide a better fit, χ²(2) = 3.68, p=0.16.

### S2.2.1 Similarity to Prior


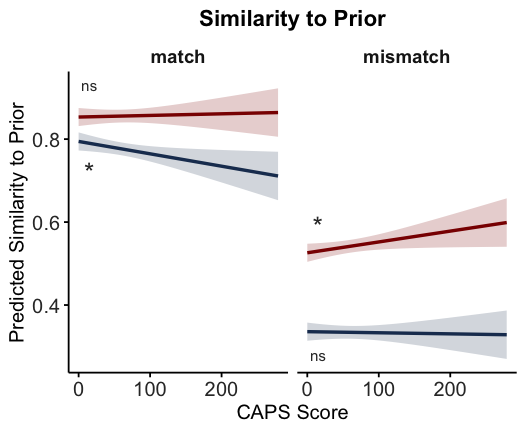

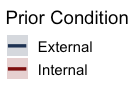


**Figure 6.** Similarity to Prior. The slope of CAPS score was significantly more negative in external-match than internal-match condition, and significantly more positive in internal-mismatch than external-mismatch condition

To test the hypothesis that similarity between word reports and priors varies with prior type and its source (internal vs external) as CAPS scores increase, we added a fixed effect of CAPS score to the model. We compared this to a more complex model which also included an interaction with clarity (CAPS score x condition x type x clarity). The more complex model did not provide a significantly better fit (AIC=—3187.2, BIC=- 3086.8) than a model without a CAPS score x clarity interaction (AIC=-3191.4, BIC=-3113.2). This was also supported by model comparisons using likelihood ratio tests which indicated that the more complex model did not provide a better fit, χ²(4) = 3.8794, p=0.4226. Therefore, we retained the following model to examine the effects of CAPS score on perceptual performance:

average similarity to prior ~ clarity* condition *type + CAPS score* condition * type + (1|participant)

Model comparisons indicated that a model with a CAPS score x condition x type interaction (AIC=—3191.4) provided a better fit for the data than a model without (AIC=-3171.8), χ²(4) = 27.579, p<0.01.

Examination of model summary output indicated that higher CAPS scores were associated with lower similarity to priors (β=-0.000299, SE=0.000131, t=-2.28, p=0.023). There was a significant interaction between condition and CAPS score (F(1,1852)=16.9266, β=-000337, SE=0.000107, t=3.14, p<0.01), and a two-way interaction between prior type and CAPS score, F(1,1852)=10.5984, p<0.01, β=0.000272, SE=0.000107, t=2.53.

To understand the nature of these interactions, we conducted simple slopes analyses of CAPS score within each level of prior type, and within each condition.

Simple Slopes Analyses

We conducted simple slopes analyses of CAPS score within each level of prior type (the moderator variable, match vs. mismatch) when condition is external and internal. Estimated slopes of CAPS score for each condition (external vs. internal) were obtained separately for each prior type (Figure 6).

When the prior was matching, the slope of CAPS score was positive but non-significant (β=0.00004, SE=0.00013, t(193)=0.29594, p=0.77) in the internal condition, and significantly negative in the external condition (β=-0.0003 SE=0.00013, t(193)=-2.28 p<0.05).

When the prior was mismatching, the slope of CAPS score was negative but non-significant in the external condition (β=-0.00003 SE=0.00013, t(193)=-0.20, p=0.84), and positive and significant in the internal condition (β=0.00026 SE=0.00013, t(193)=1.99, p<0.05).

These results suggest that as CAPS scores increase, word reports are more similar to an internally generated prior when it mismatches the subsequent stimuli, and less similar to an externally provided prior which matches the subsequently presented stimuli.

Slope comparisons

To formally test whether CAPS score slopes differed between internal and external conditions within each prior type, we conducted pairwise comparisons of simple slopes using estimated marginal trends.

The difference in the slopes between external and internal conditions was significant for both matching, β=-0.000337, SE=0.000107, t(1852)=-3.140, p=0.0017, and mismatching, β=-0.000288, SE=0.000107, t(1852)=-2.678, p=0.0075, prior types, suggesting that when the prior matched the subsequently presented stimuli (i.e., it was a correct cue), as CAPS score increased, participants relied less on priors provided from an external source compared to those generated internally. In contrast, as CAPS score increased, participants’ guesses were more similar to their prior when this prior was incorrect (i.e., it mismatched the subsequent stimuli), and generated internally, compared to when it was provided externally.

### S2.2.2 Shift Index

To understand the nature of the CAPS score-by-prior-condition interaction, we compared estimated marginal means (EMMs) of the shift index between the two conditions at multiple levels of CAPS score, adjusting for multiple comparisons.

Pairwise comparisons showed that the shift index was significantly lower in the internal condition than the external condition, i.e., participants’ guesses were more similar to their priors than stimuli played, at both low (β=-0.234, SE=0.0132, t(878)=-17.767, p<0.00001) and high (β=-0.274, SE=0.012, t(878)=-23.12, p<0.0001) CAPS scores.

Pairwise comparisons between high and low CAPS scores within each condition revealed that the shift index was significantly lower in participants with high CAPS scores in the internal condition, (β=-0.050 SE=0.0196, t(127)=-2.552, p<0.05), i.e., the extent to which their guesses were influenced by their priors when those priors were generated by participants themselves and did not match the subsequent stimuli was greater when CAPS scores were higher. This association was not significant when the priors were provided to the participants externally, (β=-0.01 SE=0.0196, t(127)=-0.534, p=0.594).

### S2.2.3 Additional visualisation of hallucination-proneness effects

To visualise how condition effects manifest at the extremes of hallucination proneness, we additionally plotted model-based estimated marginal means from models refit in participants scoring at or below the 10^th^ percentile and at or above the 90^th^ percentile of CAPS, for report accuracy (Figure 7, left panel) and the shift index (Figure 7, right panel). These analyses are intended for descriptive purposes and do not replace the continuous CAPS analyses reported in the main paper.


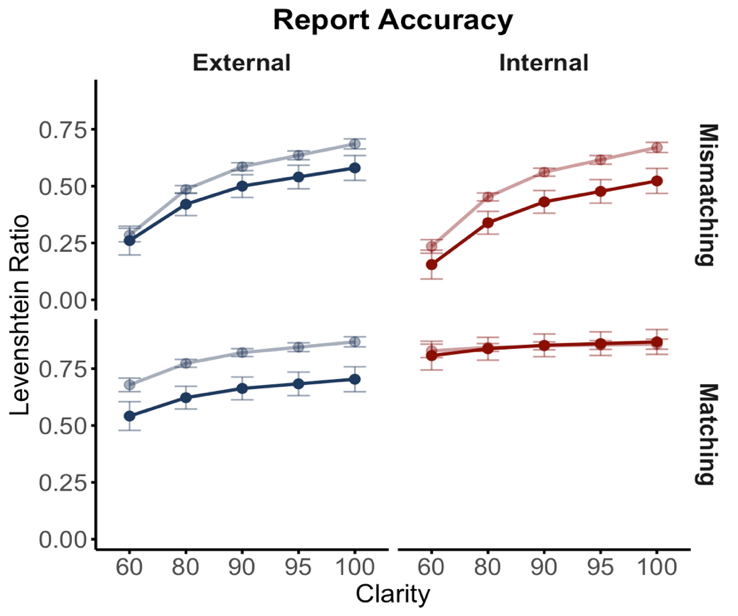

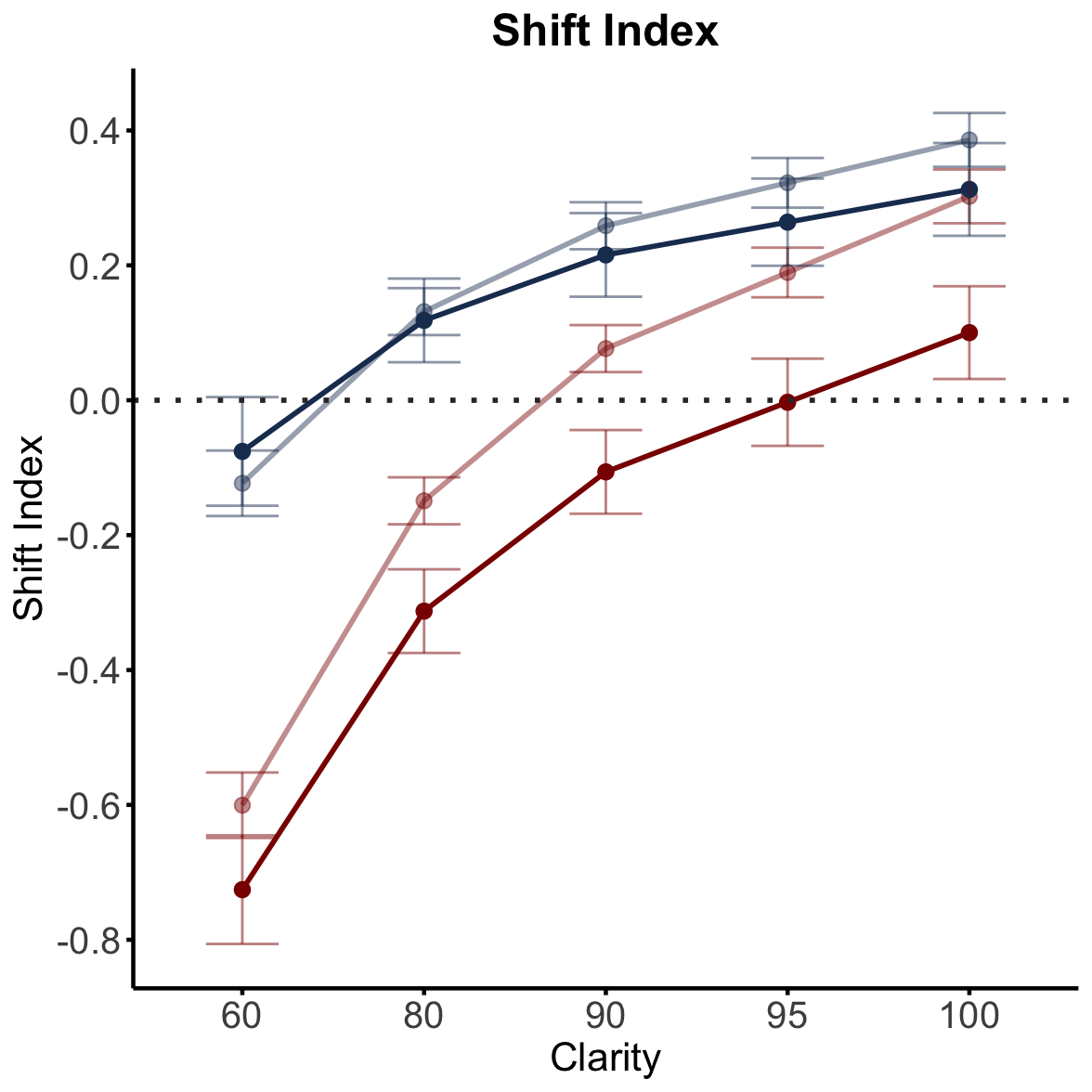

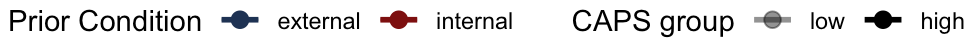


**FIGURE 7. Left:** Report accuracy for participants in the low group (CAPS ≤ 10^th^ percentile, CAPS = 0) and high group (CAPS ≥90^th^ percentile, CAPS ≥17). **Right:** Shift index for the same low and high CAPS groups.

## S2.3 Distribution of CAPS and PDI scores

PDI scores had a mean of 38.0 (SD=36.6) and a median of 31. CAPS and PDI scores were positively correlated (Pearson’s r=0.805, t=13.279, p<.01, Figure 8A). The distributions of CAPS scores differed between the two experiments (Figure 8B), likely due to slight differences in exclusion criteria between the two studies (in Experiment 2, a self-reported history of mental health problems was not used as an exclusion criterion). Levene’s test indicated significant differences in the CAPS score variances between the two experiments, F(1,188) =7.0074 , p<0.01, and the Kolmogorov-Smirnov test revealed significant differences in the overall distribution shapes, D=0.2134, p=0.0184.

**
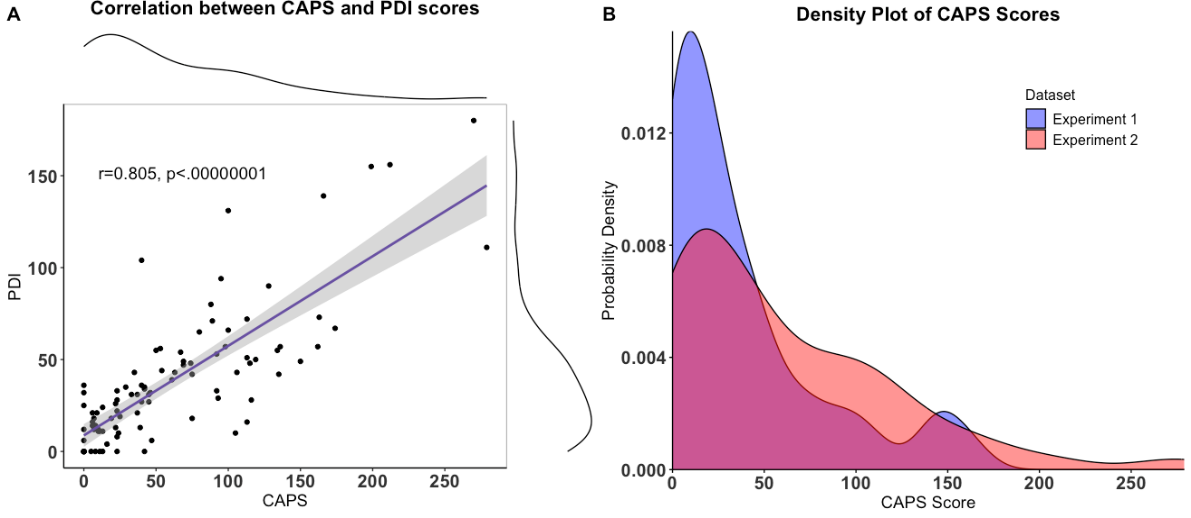
**

**FIGURE 8. A)** Proneness to hallucinations (CAPS) and delusions (PDI) were significantly correlated (N=98). **B)** Kernel density estimates of CAPS scores for both experiments showing differences in CAPS score distributions between the two experiments.

CAPS scores in our sample fell within the lower-to-mid range of published population distributions. In Bell et al. (2011), the normative mean was 7.41 (SD=6.24). Experiment 1 showed a mean of 4.70 (median 3, 30^th^ percentile), and Experiment 2 a mean of 6.60 (median = 5, 40-50^th^ percentile). As we did not pre-screen for high proneness, very high scores (e.g. ≥90^th^ percentile) were rare.

## S2.4 Robustness checks

We were encouraged by a reviewer to explore the robustness of our effects by considering other means of parameterising high/low CAPS differences. In Experiment 1, CAPS-related reductions in perceptual sensitivity (d’) are consistently robust, appearing under multiple analytic specifications of high/low CAPS individuals and stable under robustness checks using leave-one-(participant)-out resampling. Criterion effects are weaker, which is expected given the small effect size, but directionally consistent. In Experiment 2, the CAPS-by-condition interaction effect showed the same pattern across alternative parameterisations (ranked and log-transformed CAPS scores), though statistical significance was attenuated for transformations that reduced variance. We are thus reassured that our core conclusions are not dependent on specific analytic choices.

# Section 3: Stimuli

## S3.1 List of spoken words

| sat | fall | wrote | well | feel |
| --- | --- | --- | --- | --- |
| role | bed | use | ten | whole |
| lord | back | red | set | wall |
| had | whose | knows | part | sign |
| bit | size | hard | not | main |
| what | paid | cut | light | made |
| side | male | court | days | head |
| right | led | call | word | weight |
| lot | done | but | when | tax |
| line | can | born | sort | six |
| cause | leave | goes | son | mass |
| board | keep | god | sight | man |
| will | has | fell | showed | like |
| bad | got | died | said | let |
| sit | does | work | ran | heart |
| road | deal | top | met | heard |
| read | white | run | make | hall |
| rate | while | room | late | fine |
| ways | walk | rest | hot | been |
| says | seen | miss | heat | talk |

**Table 1.** Words used as stimuli
